# Tailored implementation of the FICUS multicomponent family support intervention in adult intensive care units: findings from a mixed methods contextual analysis

**DOI:** 10.1186/s12913-023-10285-1

**Published:** 2023-12-01

**Authors:** Lotte Verweij, Saskia Oesch, Rahel Naef

**Affiliations:** 1 Institute for Implementation Science in Health Care, Faculty of Medicine, University of Zurich, Zurich, Switzerland; 2 Centre of Clinical Nursing Science, University Hospital Zurich, Zurich, Switzerland

**Keywords:** Implementation science, Tailored implementation, Contextual analysis, Mixed methods, Family support intervention, Critical care

## Abstract

**Background:**

The Family in Intensive Care UnitS (FICUS) trial investigates the clinical effectiveness of a multicomponent, nurse-led interprofessional family support intervention (FSI) and explores its implementation in intensive care units (ICUs). The local context of each ICU strongly influences intervention performance in practice. To promote FSI uptake and to reduce variation in intervention delivery, we aimed to develop tailored implementation strategies.

**Methods:**

A mixed method contextual analysis guided by the Consolidated Framework for Implementation Research (CFIR) was performed from March to June 2022 on eight ICUs assigned to the intervention group. ICU key clinical partners were asked to complete a questionnaire on CFIR inner setting measures (i.e., organizational culture, resources, learning climate and leadership engagement) and the Organizational Readiness for Implementing Change (ORIC) scale prior to group interviews, which were held to discuss barriers and facilitators to FSI implementation. Descriptive analysis and pragmatic rapid thematic analysis were used. Then, tailored implementation strategies were developed for each ICU.

**Results:**

In total, 33 key clinical partners returned the questionnaire and 40 attended eight group interviews. Results showed a supportive environment, with CFIR inner setting and ORIC measures each rated above 3 (scale: 1 low—5 high value), with leadership engagement scoring highest (median 4.00, IQR 0.38). Interview data showed that the ICU teams were highly motivated and committed to implementing the FSI. They reported limited resources, new interprofessional information exchange, and role adoption of nurses as challenging.

**Conclusion:**

We found that important pre-conditions for FSI implementation, such as leadership support, a supportive team culture, and a good learning climate were present. Some aspects, such as available resources, interprofessional collaboration and family nurses’ role adoption were of concern and needed attention. An initial set of implementation strategies were relevant to all ICUs, but some additions and adaptation to local needs were required. Multi-component interventions are challenging to implement within complex systems, such as ICUs. This pragmatic, theory-guided, mixed methods contextual analysis demonstrated high readiness and commitment to FSI implementation in the context of a clinical trial and enabled the specification of a tailored, multifaceted implementation strategy.

**Supplementary Information:**

The online version contains supplementary material available at 10.1186/s12913-023-10285-1.

## Background

Family members of critically ill patients treated in intensive care units (ICUs) experience high levels of stress and uncertainty, which often result in adverse post-ICU mental health outcomes, such as anxiety, depression, and post-traumatic stress [1–3], also known as Post-Intensive Care Syndrome-Family (PICS-F) [4]. Better support and communication structures for families experiencing critical illness has therefore been called for, but research remains inconclusive about their clinical effectiveness [4, 5]. To address the lack of research on the effectiveness and successful implementation of interventions focusing on families’ needs, we developed, piloted, and are currently testing a multi-component family support intervention (FSI) [6–8].

The implementation of complex interventions in health care settings is considered challenging [9–11]. The dynamic contextual determinants within ICUs are highly likely to impact the FSI uptake and delivery [12–14] and could consequently influence the degree of effectiveness [15–18]. To ensure successful implementation of complex interventions, tailoring of implementation strategies to the local context is considered a key factor [17, 19, 20].

Contextual determinants are defined as factors believed or empirically shown to influence the implementation process and thereby implementation and intervention outcomes [9, 21–23]. Research has identified organizational, team, staff, and family-related determinants to shape the uptake of evidence-based practices in routine care delivery [24–27]. For example, at the organizational level, leadership and organizational support, physical layouts, staffing levels, and time capacity are key determinants to family care and frequent barriers [28–32]. At the team level, interprofessional team commitment and established care structures, such as regular family meetings or open visitation policies for families, were consistently identified as enablers, whereas perceptions of families as interrupters coupled with lower educational levels pose barriers [28, 30, 32–34]. At the family level, language, health literacy, complex or conflictual family structures and dynamics, together with patient acuity, hinder the uptake of evidence-based family care processes [25, 30–32]. In terms of the implementation into routine care, opportunities for mutual and ongoing learning, teambuilding, interprofessional commitment, and use of champions were found to be enablers in the uptake of evidence-based family engagement practices, coupled with organizational readiness, available resources, leadership support and high energy levels of staff [26, 35].

Knowledge of contextual determinants and implementation processes in the context of clinical trials testing FSIs in ICUs is lacking to date [36–41]. Research insights into contextual determinants and tailored implementation in FSI research in ICUs has yet to be provided.

The Family in Intensive Care UnitS (FICUS) Family Support Intervention (FSI) entails three components that are delivered to families by ICU-certified family nurses who work in close collaboration with the interprofessional team along the patient pathway, including follow-up; [1] nurse-coordinated liaison, [2] therapeutic family conversations, and [3] structured, interprofessional communication [6]. The FSI was then implemented on one ICU and pilot-feasibility tested in a before – after study with process evaluation. The results showed that families receiving nurse-led FSI experienced increased satisfaction with care and an even more significant increase in satisfaction with the decision-making process [8]. Families and ICU staff considered the FSI to be an acceptable, appreciated, and beneficial model of care [7]. Based on these promising results, the FSI was refined and scaled-up for implementation and evaluation of its clinical effectiveness in the FICUS cluster hybrid Randomized Controlled Trial (RCT) [6]. As recommended in implementation science, a clear delineation of implementation strategies in distinction to the intervention is necessary [42, 43]. Therefore, based on the findings of the pilot-feasibility study, a set of implementation strategies was predefined, including ICU leadership endorsement, family nurse interventionist training with monthly case conferences and individual coaching and support, ICU team education about the FSI, nurse and physician champions, and local and central implementation facilitators [6].

Contextual analyses are a helpful tool to identify determinants in implementation processes and consequently to adapt and tailor the intervention and implementation strategies to the specific features of the context [44–46]. Context is defined as any feature of the circumstances in which an intervention is conceived, developed, implemented, and evaluated [9, 21]. To support the implementation process of the FSI and to reduce variation in intervention delivery, the current study aimed to identify context-specific needs for implementation and to develop a tailored implementation strategy to support the FSI in the ICUs participating in the FICUS trial.

## Methods

### Design

This study is part of a hybrid effectiveness-implementation study [47, 48] and embedded within the FICUS multicenter cluster RCT [6]. To tailor the pre-defined implementation strategy to specific contextual needs, we performed a mixed methods contextual analysis in the eight intervention ICUs [49]. The contextual analysis included a questionnaire consisting of validated self-reported instruments that assess contextual determinants to implementation at the organizational level, followed by eight group interviews. Reporting of the study followed the GRAMMS checklist for the Good Reporting of A Mixed Methods study [50], see Supplementary File 1.

### Framework

To systematically assess contextual determinants (barriers and facilitators) in the implementation of the FSI, the Consolidated Framework for Implementation Research (CFIR) was used [51]. The CFIR is a theory-based determinant framework and comprises five interrelating major domains (intervention characteristics, outer setting, inner setting, characteristics of individuals and the process of implementation) with 37 constructs relevant to implementation. These constructs can each act as a barrier or facilitator within the implementation process. The questionnaires focused mainly on the CFIR inner setting domain, whereas the interview topics covered all CFIR constructs.

We combined the CFIR framework with the Expert Recommendation for Implementation Change (ERIC) strategy tool in refining the planned implementation strategy for each of the participating ICUs [19, 52]. The ERIC tool has been developed to address implementation barriers identified according to the CFIR. The tool includes 73 implementation strategies, which are clustered into nine domains of implementation recommendations, i.e.: 1) use evaluative and iterative strategies, 2) adapt and tailor to context, 3) train and educate stakeholders, 4) engage consumers, 5) change infrastructure, 6) provide interactive assistance, 7) develop stakeholder interrelationships, 8) support clinicians, and 9) utilize financial strategies.

### Setting

The contextual analysis took place in the eight FICUS intervention ICUs in the German speaking part of Switzerland [6]. The included ICUs are located in two university-affiliated, three cantonal, two regional and one private hospitals and run 8 to 20 beds.

### Participants

Participants were health care professionals i.e., key clinical partners from the eight ICUs, with a relevant role in the implementation process or delivery of the FSI intervention. A purposive sampling method was used to recruit family nurses who delivered the FSI intervention, persons taking on a role of implementation support, as well as ICU nurses and physicians with a leadership position.

### Data collection

Data collection took place from March to June 2022, prior to trial start. The FSI local implementers were asked to plan and coordinate the interview date and time with the local staff. Two weeks prior to the group interviews, a questionnaire was sent by email to the key clinical partners with the request to return it at least two days prior to the interview date. The questionnaire could be completed digitally or in print (return as scan).

#### Questionnaire

From each key clinical partner, years of work experience, years working at the ICU, their profession and if the person is in a leadership position, was collected.

To assess organizational influences, 34 items of six CFIR inner setting constructs were used, consisting of [1] ‘culture overall’ – 9 items, defined as ‘norms, values, and basic assumptions of a given organization’, [2] ‘culture stress’ – 4 items, defined as ‘perceived strain, stress and role overload’, [3] ‘culture effort’ – 5 items, defined as ‘how hard people in organizations work towards achieving goals’, [4] ‘learning climate’ – 5 items, defined as ‘a climate in which team members feel valued and needed, feel safe and feel time and space for evaluation and, [5] ‘leadership engagement’ – 4 items defined as ‘commitment, involvement, and accountability of leaders and managers’, and [6] ‘available resources’ – 7 items, defined as ‘the level of resources dedicated for implementation and ongoing operations including money, training, education, physical space, and time [53]. The internal consistency of the sub-constructs showed Cronbach’s alpha for culture overall = 0.89, culture stress = 0.85, culture effort = 0.79, learning climate = 0.85, leadership engagement = 0.92 and available resources = 0.81, in the original English language version. With permission of the authors, using a forward–backward translation procedure [54], the questionnaire was translated from English into German. Three independent translators were involved in the procedure. The CFIR inner setting questionnaire uses a 5-point Likert scale, varying from 1 – strongly disagree to 5 – strongly agree. Higher scores indicate a more supportive implementation context.

To assess organizational readiness, the German version of the 12-item Organizational Readiness for Implementing Change (ORIC) questionnaire was used [55]. Organizational readiness is defined as ‘the extent to which organizational members are psychologically and behaviorally prepared to implement organizational change [56]. The questionnaire is based on Weiner’s theory of organizational readiness for change in health care settings [57], and includes two subscales, i.e., ‘change commitment’ and ‘change efficacy’, defined as organizational members’ shared resolve to implement a change and organizational members’ shared belief in their collective capability to implement a change [57, 58]. Cronbach’s alpha showed 0.92 for change commitment and 0.88 for change efficacy in the English language version [55]. Items are rated on a 5-point Likert scale, varying from 1 – disagree to 5 – agree. Higher scores indicate that members are more likely to initiate change, exert greater effort, exhibit greater persistence, and display more cooperative behavior, resulting in more effective implementation [58].

#### Group interviews

Eight group interviews with two to eight key clinical partners were conducted by two researchers (SO and LV) who alternated in the role of moderator and observer per interview. Interviews were organized on-site and planned for a maximum of 90 min. Group composition varied per ICU. The observing researcher took field-notes, observed non-verbal reactions, and asked for clarification if aspects were unclear. As part of our pragmatic rapid analysis approach, the field-notes served as raw data [59].

A group interview format was chosen, varying between small group interviews and focus group interviews, depending on the number of participants whereas the number of participants was expected to vary across ICUs [60]. A group format was used to enable discussion about the barriers and facilitators to the FSI and to learn from the different professional perspectives about the current practices and how they planned to integrate the FSI into care delivery. This enabled a dialogue about values, norms, expected challenges, and required actions [60].

A semi-structured CFIR-based interview guide was used to identify relevant contextual determinants (see Supplementary File 2). The interview was started by discussing the results of the questionnaires. Topics such as the need for adaptations within ICU processes to enable FSI implementation, team acceptance of the FSI and the need for specific implementation strategies were discussed. Based on the discussed barriers and facilitators, implementation strategies were jointly discussed and prioritized, and subsequently formulated in an individualized, tailored implementation plan for the first implementation phase.

### Data analysis

Data from the questionnaires were entered in and analyzed using IBM SPSS Statistics Version 28 (SPSS Inc., Chicago, IL, USA). Categorical data were summarized as frequencies and percentages, and ordinal data as medians and interquartile ranges (IQR).

For the analysis of the qualitative data, a pragmatic deductive rapid framework analysis approach was used [59]. This method is particularly suitable when there is a need for quick results that enables the development of a determinant-based tailored implementation strategy for each ICU while still ensuring a systematic data analysis process [61, 62].

The collected field-notes were read by two researchers (SO and LV) individually to get familiar with the data. Then, both researchers individually segmented the notes into the five CFIR domains and subsequently discussed the resulting allocation and influence. In the next phase, the researchers classified the notes and quotes into the CFIR sub-constructs and formed meaning units, which were thereafter discussed with a third researcher (RN) and followed by a new round of refinement. Lastly, the three researchers discussed the interpretation and classification of the data within the CFIR and finalized the data interpretation process.

The quantitative and qualitative evaluation was linked during data collection and then combined by integrating comparing and contrasting results [63]. Three researchers (SO, LV, RN) discussed and interpreted the meaning of the qualitative themes in relation to the quantitative phenomena and illustrated them graphically.

### Rigor

To ensure qualitative rigor, we applied several strategies within the different phases of the study [64, 65]. To ensure a broad perspective of ICU clinicians on the implementation of the FSI, we purposively selected clinicians with close involvement in the FSI for the group interviews. Two trained and experienced researchers (LV and SO) performed the interviews and collected the data. Due to the immediate need for tailored implementation plans after the interview, member checking in the form of a protocol and implementation plan within the days after the interview, took place. Based on clinicians feedback the protocol and implementation plans were adjusted and completed and ensured accurate interpretation of the interviews. The data analysis process was performed by three researchers (LV, SO and RN). The involvement of RN in the analysis process, who was not present during the interviews, ensured objective interpretation of the data. Data were accurately collected and securely stored and managed.

## Results

### Participant characteristics

In total, 40 key clinical partners attended the group interviews with a median of five (min. 2 – max. 8) persons per interview. During all interviews, at least the local implementer and one family nurse were present. In six of eight interviews an ICU nurse team leader and an ICU physician leader participated. Other participants included co-team leaders and clinical nurse specialists.

Of those attending the group interviews, seven (five nurses and two physicians) did not return the questionnaire (see Table 1). The median years of work experience was 22 (IQR 12.5) and a median of12 working years (IQR 15.0) at the current ICU.

**Table 1:** Participant characteristics

	Questionnaire returned *n* = 33	Questionnaire not returned *n* = 7
Nurse, n (%)	29 (88)	5 (71)
Physician, n (%)	4 (12)	2 (29)
Leadership position, number (%)	20 (61)	5 (71)
Years of work experience, median [IQR]	22 [12.5]	Not available
Years working at ICU, median [IQR]	12 [15.0]	Not available

*Abbreviations*: *IQR* Interquartile range

### Quantitative findings on organizational determinants

**Fig. 1:**
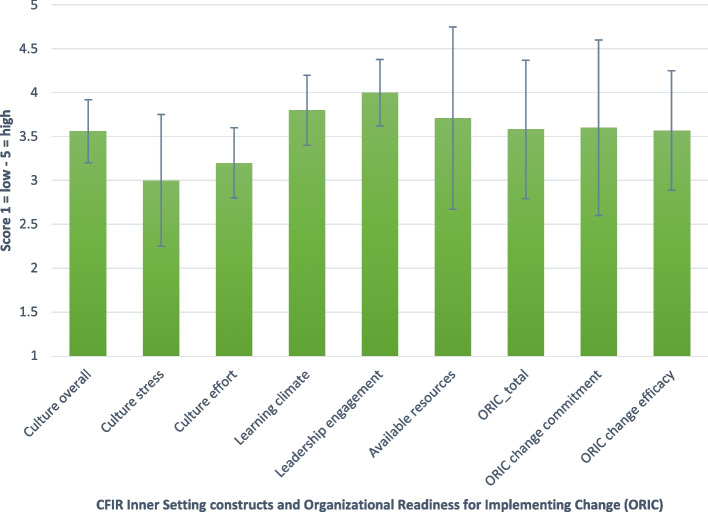
Organizational determinants to FSI implementation Legend: Values are presented in medians and interquartile ranges

The results showed that ICUs’ leadership engagement was valued highest with a median of 4.00, IQR 0.38 indicating a high level of experienced leadership support, see Fig. 1. This was followed by the ICUs’ learning climate (median 3.80, IQR 0.40), available resources (median 3.71, IQR 1.04), culture overall (median 3.56, IQR 0.36), and culture effort (median 3.20, IQR 0.40). Culture stress received the lowest score with a median of 3.00, IQR 0.75, indicating a moderate level of experienced work-related stress and workload.

The ORIC questionnaire had a median score of 3.58, IQR 0.79, indicating moderate to high perceived organizational readiness. Subscales of change commitment and change efficacy showed medians of 3.60, IQR 1.00 and 3.57, IQR 0.68, respectively. Supplementary File 3 shows the results for the single items of the questionnaire.

### Qualitative findings on contextual determinants

Results from the group interviews are presented according to the five CFIR domains. Within each domain, the constructs that were reflected in the data are delineated including barriers and facilitators to the FSI implementation. A summary of the findings is presented in Table 2.

**Table 2:** Themes of the contextual determinants according to CFIR domains and constructs [51]

**CFIR Domain with definition**	**CFIR Construct with definition**	**Theme of contextual determinants**
Intervention characteristics “Characteristics and adaptability of the intervention being implemented”	Adaptability “The degree to which an intervention can be adapted, tailored, refined, or reinvented to meet local needs.”	Interprofessional communication components of the FSI
	Relative advantage “Stakeholders’ perception of the advantage of implementing the intervention versus an alternative solution.”	Family nurses’ co-moderating role during interprofessional family meetings
Outer Setting “The economic, political, and social contexts within an organization resides”	Needs and resources of those served by the organization “The extent to which patient needs, as well as barriers and facilitators to meet those needs are accurately known and prioritized by the organization.”	Recruitment of family members and timely FSI delivery
Inner Setting “The structural, political and cultural contexts through which the implementation process will proceed”	Networks and communications “The nature and quality of webs of social networks and the nature and quality of formal and informal communications within an organization.”	Research team support
	Culture “Norms, values, and basic assumptions of a given organization.”	High value of family engagement and support
	Relative priority “Individuals’ shared perception of the importance of the implementation within the organization.”	Team motivation for change
	Available resources “The level of resources dedicated for implementation and on-going operations including money, training, education, physical space, and time.”	Concerns about staff capacity
	Access to knowledge and information “Ease of access to digestible information and knowledge about the intervention and how to incorporate it into work tasks.”	FSI training
Characteristics of individuals “Individuals defined as intervention users and other potentially affected individuals”	Self-efficacy “Individual belief in their own capabilities to execute courses of action to achieve implementation goals.”	Family nurses’ role adoption
	Other personal attributes “A broad construct to include other personal traits such as tolerance of ambiguity, intellectual ability, motivation, values, competence, capacity, and learning style.”	Time investment
Implementation process “The process of change aimed at achieving that the intervention is used at the individual and organizational level as it was designed”	Formally appointed local implementation leaders “Individuals from within the organization who have been formally appointed with responsibility for implementing an intervention as coordinator, project manager, team leader, or other similar role.”	Implementation leadership
	Engaging “Attracting and involving appropriate individuals in the implementation and use of the intervention through a combined strategy of social marketing, education, role modeling, training, and other similar activities.”	Involvement of stakeholders

### CFIR intervention characteristics

During the interviews, two themes emerged concerning the adaptability of the interprofessional collaboration and communication component of the FSI, and the relative advantage of family nurses’ co-moderating role in interprofessional family meetings.

- ➣ Adaptability—*Interprofessional communication component of the FSI.*

The integration of the FSI’s interprofessional communication structures (co-moderated family meetings, coordination, and documentation of family care), into the ICU structures was expected to be challenging. Concerns pertained mostly to logistics, such as the planning of the meeting, where and when to document family meetings to make it visible for all involved professionals as—nurses and physicians did not necessarily have access to each other’s clinical records. Despites these concerns, a need for clear and transparent interprofessional collaboration was acknowledged, and key clinical partners agreed on the need for adapted, collaborative work processes in caring for families to optimize the continuity of care.

- ➣ Relative advantage—*Family nurses’ co-moderating role during interprofessional family meetings*.

Some physicians expressed concerns about the new family nurses’ co-moderating role during the interprofessional meetings. In their view, families need is to get updated about the medical situation of their close other. Therefore, they felt a more active co-moderating role of the family nurse as interfering with this need. In some of the participating ICUs, nurses already had an established role during interprofessional family meetings. Medical staff of the latter ICUs saw benefit in nurses’ co-moderation. However, they did not see a need for change. Consequently, from a physician’s point of view, a need for the FSI interprofessional communication structure in the form of regular, co-moderated family meetings was not necessarily given.

### CFIR outer setting

Concerns were expressed about families’ needs, their willingness to participate and the timely delivery of the FSI at the time of study recruitment.

- ➣ Needs and resources of those served by the organization—*Recruitment of family members and timely FSI delivery.*

From key clinical partners’ perspective, families’ high stress level and burden following patient’s ICU admission arose as a key barrier to the ability to recruit them into the study and deliver the required intervention dose within the first four days following admission. They were apprehensive about their ability to achieve recruitment, informed consent, data collection and intervention delivery within the protocolized timeframe.

### CFIR inner setting

The raised issues pertained to five constructs or sub-constructs of the inner setting domain.

- ➣ Networks and Communications – *Research team support.*

For most of the participating units and key clinical partners, participating in a clinical research project was a new experience. Participants expressed the need for and importance of the research team's support to guide them throughout the implementation process in the ICUs.

- ➣ Culture – *High value of family engagement and support.*

Family engagement and support is highly valued by most ICU teams according to key clinical partners. This is an important precondition for the implementation process and acceptance of the intervention within the ICU teams.

- ➣ Relative priority – *Team motivation for change.*

From key clinical partners’ perspective, nursing teams did not always express optimism towards the upcoming change. They explained this as an expression of ‘innovation exhaustion’ of the teams, which was in their view a result of the high workload during and after the COVID-19 pandemic. However, the teams did see a need for change and key clinical partners had not experienced reluctance regarding the FSI within the nursing teams. Open and transparent communication about the study and a careful implementation process were considered key to motivate nursing teams and to ensure a supportive implementation climate.

- ➣ Available resources – *Concerns about staff capacity.*

From an ICU management perspective, patient care has absolute priority. Since the COVID-19 pandemic, ICU leaders have experienced a high rate of absenteeism and exhaustion among nursing staff. Team leaders expressed their concerns about a potential conflicting situation between primary patient care-related and study intervention-related tasks. They had limited possibility to find additional staff for the FSI or staff to replace those involved in the FSI, despite available financial resources. Notwithstanding these worries regarding staff capacity impacting negatively on their readiness for implementation, ICU leaders acknowledged the importance of study participation and appraised the participation of staff in the FSI as an opportunity for professional development. Furthermore, they expressed that a clear understanding of the roles and tasks associated with the FSI promotes efficient collaboration and work processes between the staff involved and was considered key for successful implementation.

- ➣ Access to knowledge and education – *FSI training.*

Skill development in the FSI and the underlying intervention theory and approach to care, such as family systems nursing through the five-day FSI course, was experienced positively by involved nurses. The theoretical knowledge and the skills training support was experienced as an important prerequisite to perform the intervention in the real-world settings of the ICUs. Also, the availability of intervention resources online was mentioned to be beneficial.

### CFIR characteristics of individuals

Family nurses expressed uncertainty in adopting their role in the context to the FSI. Physicians expressed concerns about a potential increase in time investment for interprofessional family meetings.

- ➣ Self-efficacy – *Family nurses’ role adoption*.

Despite the five-day training course, participating family nurses experienced their new role within the FSI as challenging. Some expressed feelings of uncertainty about performing their new role in practice. They lacked a role model and struggled to imagine what the intervention would look like in practice. They considered the planned monthly case conferences as an important opportunity to develop their skills through reflection and mutual learning among peers involved in delivering the FSI and considered this an important resource for implementation.

- ➣ Other personal attributes – *Time investment.*

Some participating physicians expressed concerns about a possibly increased need for interprofessional family meetings as a part of the FSI. With the family nurses initiating these meetings based on their assessment of the families' situations and needs, physicians expressed that this could get complicated to fit into their already tight schedule.

### CFIR Implementation process

Interview participants appreciated the opportunity to be involved in the planning of the FSI implementation. They valued the role of a formally appointed local implementer for the FSI and emphasized the importance of engaging nursing and physician team leaders in the local implementation process.

- ➣ Formally appointed local implementation leaders – *Implementation leadership.*

Key clinical partners expressed the importance and valued the appointment of a local implementer to the translation of the FSI into practice, to bridging between the research team and the local staff involved in the FSI, and to communicating about the FSI within the local teams. In addition, they mentioned that knowledge about the local culture and organizational structures is an important precondition to perform this task. For instance, knowledge about informal leaders within the teams was mentioned to be of importance.

- ➣ Engaging – *Involvement of stakeholders.*

The involvement of nursing team leaders and physicians as partners in the implementation process was considered essential for all ICUs. They had already made the experience that by their involvement, the integration process and acceptance by teams could be raised and potential reluctance within teams targeted early on. Nursing team leaders and physicians were not involved as stakeholders in all ICUs. Other stakeholders such as palliative and social care teams were considered as important stakeholders.

### Integrated findings on organizational determinants

**Fig. 2:**
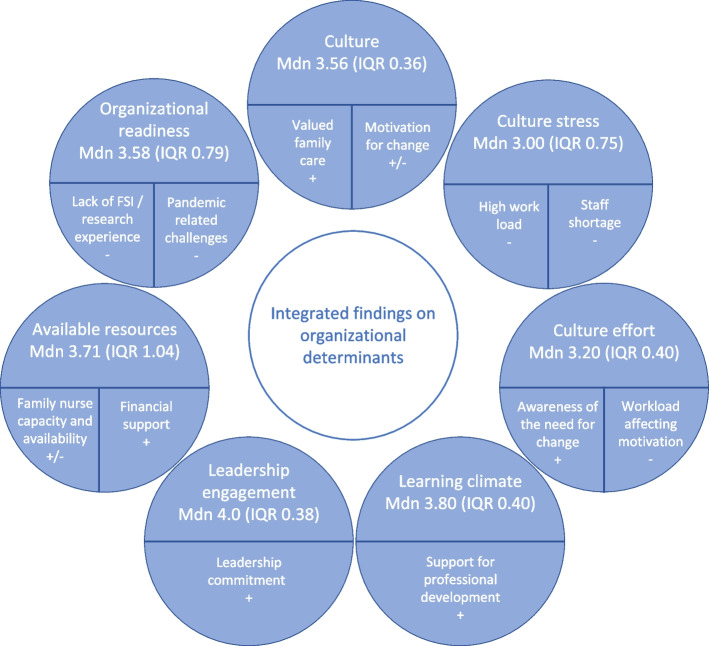
Integrated findings on organizational determinants Legend: upper half – CFIR inner setting constructs, Mdn Median, IQR interquartile Range, lower half – qualitative findings within CFIR inner setting constructs. + facilitating,—hindering, ± mixed influence at organizational level

Qualitative findings on the organizational determinants provide a more nuanced understanding of the quantitative findings. In Fig. 2, we present the integrated findings by reporting the quantitative findings in the upper half and the explainable qualitative findings in the lower half. Some qualitative findings, i.e. motivation for change and family nurse capacity and availability, were experienced supportive as well as hindering by ICUs.

### Tailoring of FSI implementation strategies

After the identification of local barriers and needs through the contextual analysis, the pre-defined FSI implementation strategy was tailored to the specific contexts and needs in close collaboration with the key clinical partners and resulted in a tailored implementation plan per ICU.

Guided by the CFIR-ERIC matching tool, the identified strategies reflected mainly the pre-defined implementation strategies, but some refinements and adaptations were needed that mainly considered the frequency or dose and the targeted group. For instance, in some ICUs, the medical staff was not yet informed to a sufficient extent about the FSI implementation. Information meetings were then prioritized and concretized with the local staff. A few additional strategies were required. For example, the engagement of physicians with a leadership role showed to be important and regular stakeholder meetings and opportunities for individual feedback were organized. The updated, multifaceted implementation strategy was then specified according to recommendations, see Table 3 [66].

**Table 3:** Refined and specified multifaceted implementation strategy

**ERIC strategy domains**	**FSI – Implementation strategies**	**Actor(s)**	**Action**	**Action targets—CFIR**	**Temporality – Dose**
Develop stakeholder interrelationships	Leadership and stakeholder engagement	Research team	Work collaboratively with local ICUs	Engaging, networks and communication, implementation climate and readiness for implementation	Ongoing
Adapt and tailor to context	Contextual analysis	Key clinical partners Research team	Identify local barriers and enables	Readiness for implementation, adaptability	Prior to study start
	Tailored implementation	Key clinical partners Research team	Act on identified (expected) barriers	Readiness for implementation, adaptability	Ongoing
Train and educate stakeholders	Intervention nurse training	ICU family nurses Research team	Educate and train family nurses in FSI delivery	Self-efficacy	Prior to study start – 5 days
	Implementation support training	Local implementer Research team	Educate and train local implementers in roll-finding and implementation support	Self-efficacy, implementation climate and readiness for implementation	Prior to study start
	Team information meetings	ICU family nurses Local implementer Research team	Inform and educate ICU team about FSI	Implementation climate and readiness for implementation	Prior to study start and upon request
Support clinicians	Individual coaching and support	Research team	Ensure consistent high quality FSI delivery and support self-efficacy	Self-efficacy, engaging	Once midterm and upon request
	Case conferences	Family nurses Research team	Ensure consistent high quality FSI delivery, support self-efficacy	Self-efficacy, engaging	Monthly during implementation
Provide interactive assistance	Local implementation support	Local implementer	Support implementation and delivery of FSI according to protocol	Implementation climate, readiness for implementation and engaging	Ongoing
	ICU leadership support	Research team	Ensure that unit leadership support implementation	Implementation climate, readiness for implementation and culture	Ongoing
	External implementation support	Research team	Support stakeholders in implementation	Self-efficacy, engaging, planning and other personal attributes	Ongoing
	Nurse and physician champions	Key clinical partners	Ensure team-based facilitation	Implementation climate, readiness for implementation, culture, engaging	Ongoing
Use evaluative and iterative strategies	Regular stakeholder meeting	Key clinical partners Research team	Meet with key clinical partners to enable ongoing adaptations to implementation	Networks and communications, implementation climate, and readiness for implementation, other personal attributes	Quarterly
	Ongoing feedback-loops	Key clinical partners Research team	Enable ongoing evaluations and room for discussion	Networks and communications, self-efficacy, planning, adaptability	Ongoing

## Discussion

In this mixed method contextual analysis, which was undertaken prior to the implementation of the FSI in ICUs within the scope of the FICUS trial, we found high leadership commitment and learning climate, adequate organizational readiness and resources, and an overall supportive culture challenged with workload and staffing pressures. Qualitative findings confirmed leadership and clinician commitment to developing family care through pragmatic research embedded in the context of ICU care. Identified barriers were mainly related to questions of adequacy for and needs of families, the required adaptations in interprofessional collaboration, nurses’ new role, concerns around staff capacity due to post-pandemic exhaustion and shortages, and availability / planning of intervention nurses. The network, training, and specific implementation support roles available through the trial arose as enablers. Early engagement and inclusion of nurses, physicians, and unit leadership was considered a key strategy for enabling implementation success. The proposed multifaceted implementation strategy for the FSI was therefore relevant but required some additions and refinement for each ICU.

This contextual analysis contributes insights into specific barriers and enablers to the implementation of complex FSIs in ICUs. Our thematic findings related to all five CFIR domains, among which inner setting constructs were represented most strongly. The combination of qualitative and quantitative insights at the level of ICUs suggests that factors such as resources, culture, priority-setting as well as leadership support and access to a wider support network play a key role in implementation [67]. These identified contextual determinants reflect findings from a recent review, which found that determinants within the organizational domain, such as cultural aspects and available resources (i.e. workload and staffing) were main barriers to implementation of family care practices, whereas team determinants such as shared understanding and commitment were found to be key facilitators [26].

Clearly specified implementation strategies that target barriers and amplify facilitators inherent to a specific environment, such an ICU, are key to ensure successful integration of complex interventions such as FSI [23, 68]. Clinical trials examining similar family interventions in ICU increasingly report on the use of specific implementation strategies to support implementation of the study intervention, such as intensive educational programs, the appointment of champions and local investigators and the engagement of leadership [36–39, 41]. These implementation strategies are well-known and frequently applied withing ICU family care [26, 35]. In addition, leadership support [69], implementation facilitation [70–73], together with capacity-building strategies [26, 74] have been found to be promising implementation strategies in general. The use of a context-adapted and clearly specified implementation approach tailored to local units and developed in collaboration with key clinical partners and users is an evidence-based recommendation from implementation science [44, 66, 75]; a strategy that needs to be increasingly applied to the field of complex family support interventions in ICU.

The contextual analysis enabled adaptations and refinements of the suggested implementation strategy based on ICU-specific circumstances and needs prior to the FICUS trial and was therefore a valuable method. The thereby developed and refined multifaceted FSI implementation strategy falls into six of the nine ERIC implementation strategy domains [75]. The ERIC taxonomy covers a wide spectrum of implementation strategies that are rather general and abstract in nature [76]. The barriers identified in our contextual analysis related to ICU as a micro-level organizational unit required concretization and specifications. The available CFIR-ERIC strategy matching tool was therefore of limited use to strategy design but was able to serve as a useful structuring and verification tool. The application of recommendations for specifying implementation strategies, however, was essential to ensure clarity, specificity, and traceability of each strategy [66, 77, 78].

This context assessment is a first step within the implementation process of the FICUS FSI. Implementation processes of new interventions within teams and into established work procedures requires intensive and continued guidance. Several change management models in health care have been developed to guide and support implementation processes such as the FSI implementation and my be useful to guide change and implementation processes over time [79, 80].

### Strengths and limitations

Our study entails some methodological considerations. First, we performed this contextual analysis with a selected group of clinicians from each ICU who have a role within the implementation of the FSI, consisting of the local implementers, family nurses, (co) team leaders, physicians and clinical nurse specialists, in various group compositions. Although these clinicians were significantly affected or directly involved in the FSI implementation, they may not have been representative for the whole ICU team. It is likely that the involved clinicians with their various backgrounds have been able to express the broader ICU team perspective on the FSI implementation, however it may be possible that we missed perspectives from individuals not involved. These could have been either supportive or reluctant towards the FSI implementation and could potentially have influenced the priority-setting of implementation strategies. Second, the presence of team leaders during interviews could potentially have influenced other participants in their willingness to openly discuss barriers within the ICU. Third, for this context assessment, we have decided to focus on the perspective of clinicians in the clinical context where the FSI was implemented in, the FSI deliverers. The perspectives of family members, the FSI receivers, were not included, even though this would have been a valuable source, family member perspectives on the FSI were already represented in the feasibility study evaluation [7]. In addition, family members collaborate with the research team at the level of the FICUS trial [6]. Fourth, we performed a pragmatic rapid analysis based on field-notes taken during the interview. Although this is an established approach for qualitative analysis within implementation science [59], we may have overlooked certain contextual determinants that could have been detected with a more thorough approach such as analysis of audio-taped and transcribed interviews. The use of a systematic approach by involving three researchers in the data collection and analysis and by sending a summary to participants for verification minimized this risk.

The use of the established CFIR [51] to guide the entire study process, complemented with the CFIR-ERIC matching tool [75] and recommendations [66] for identifying, structuring and reporting our multifaceted implementation strategy, represents a clear strength. The use of a mixed method design with integration during data collection and analysis enabled us to gain in-depth understanding of ICU contexts and informed a nuanced implementation approach [46, 49]. The ICUs included in our study cover a broad range of clinical specialties and represent the entire spectrum from smaller ICUs to larger ICUs, regional centers and academic hospitals. It remains unclear, however, how representative these ICUs are of the Swiss and other cultural contexts, as it may well be that ICUs agreeing to take part in the trial may exhibit greater readiness than others.

## Conclusion

Our study provides insights into contextual determinants to complex FSI implementation and ICU family care and provides insights into types of tailored strategies that are perceived as promising by ICU clinicians. The methodology may serve as a template for the planning and designing of future implementation efforts in ICU family care and clinical trials of family support interventions. Our findings point to the key role of collaborative team processes, supportive organizational structures, and sufficient capacity to the implementation of complex family support interventions in the context of a pragmatic clinical trial. Together with an evolving body of research on implementation of family care in ICUs, our findings suggest the need to target interactive and structural determinants in ICUs. Evaluation of implementation strategies is needed to build a body of knowledge on effective implementation of family support in ICUs. The actual potential and ability of our specified, multi-faceted implementation strategy to successfully integrate the FSI in each ICU within the scope of the FICUS trial will be carefully evaluated (https://osf.io/8t2ud).

### Supplementary Information

**Additional file 1.** **Supplementary File 1.** GRAMM Checklist.

**Additional file 2.** **Supplementary File 2.** Interview guide context assessment – Implementation of the FICUS intervention.

**Additional file 3.** **Supplementary File 3.** Single items of the questionnaire.

## Data Availability

The dataset used and analyzed during the current study are available from the corresponding author on reasonable request.

## Abbreviations

CFIR: Consolidated Framework for Implementation Research
ERIC: Expert Recommendations for Implementing Change
FICUS: Family Support Intervention in Intensive Care Units
FSI: Family Support Intervention
ICU: Intensive Care Unit
ORIC: Organizational Readiness for Implementing Change
PICS-F: Post Intensive Care Syndrome Family
RCT: Randomized Controlled Trial
