# Supplementary material for: Tailored implementation of the FICUS multicomponent family support intervention in adult intensive care units: findings from a mixed methods contextual analysis
Source: BMC Health Serv Res. 2023 Dec 1;23:1339. doi: 10.1186/s12913-023-10285-1 (PMC10693161; doi:10.1186/s12913-023-10285-1)
Supplement: Supplementary file 1 — Additional file 1. Supplementary File 1. GRAMM Checklist. [file 12913_2023_10285_MOESM1_ESM.docx]

**Supplementary File 1. GRAMM Checklist**

| **Guideline** | **Section: Page** |
| --- | --- |
| Describe the justification for using a mixed methods approach to the research question | Methods- Design: 8 |
| Describe the design in terms of the purpose, priority and sequence of methods | Methods- Design: 8, Setting: 9, Participants: 10 |
| Describe each method in terms of sampling, data collection and analysis | Methods- Data collection: 10-13, Data analysis: 13, 14 |
| Describe where integration has occurred, how it has occurred and who has participated in it | Data analysis: 14 |
| Describe any limitation of one method associated with the present of the other method | Discussion: 29, 30 |
| Describe any insights gained from mixing or integrating methods | Discussion: 27-30 |
