# Supplementary material for: Tailored implementation of the FICUS multicomponent family support intervention in adult intensive care units: findings from a mixed methods contextual analysis
Source: BMC Health Serv Res. 2023 Dec 1;23:1339. doi: 10.1186/s12913-023-10285-1 (PMC10693161; doi:10.1186/s12913-023-10285-1)
Supplement: Supplementary file 2 — Additional file 2. Supplementary File 2. Interview guide context assessment – Implementation of the FICUS intervention. [file 12913_2023_10285_MOESM2_ESM.docx]

**Supplementary File 2. Interview guide context assessment – Implementation of the FICUS intervention**

| **Cluster ID** |  | |
| --- | --- | --- |
| **Date and time context assessment** |  | |
| **Participants** |  | |
| **Aim** | - Assess potential opportunities and challenges in the implementation of the FICUS intervention - Develop an implementation plan aligned with the IPS, including strategies for successful implementation | |
| **Preperation** | Questionnaires | |
| **Procedure** | | |
| 1. **Welcome** | a) Thanks for participation and appointment organization  b) Today we want to understand how the processes in the ICU work and where the possible difficulties and risks for the implementation of the FICUS intervention are, in order to decide on a targeted implementation strategy.  c) Important is it to give your personal assessment of the situation on the ICU  d) Discussion of topics in terms of; what are the issues that may impact the implementation of the new intervention, based on questionnaire insights and additional issues?  e) Joint identification of the top 3 supporting and inhibiting factors on the IPS. | |
| 1. **Summary of the questionnaires filled out prior to the interview** | Thank you for filling out the questionnaire. This is important information for us.  As we have understood it…  Do you agree with these statements? If yes/no why?  Regarding the FICUS study, some aspects have been answered very differently, how can we interpret these aspects?  1) How do you assess the readiness and motivation of the team to implement the FICUS intervention?  2) How do you think the interprofessional team/physician colleagues are ready to use and implement the intervention? | |
| 1. **Personal assessment** | - We would like to find out what you think will be the three biggest challenges but also the three biggest supporters/resources in the implementation of the FICUS intervention. - What factors might be present due to the pandemic?   *Assignment: we ask you to discuss these with each other and write them down on A3 and at the same time think about what could be possible strategies?* | |
| 1. **CFIR Barrier Assessment Tool** | | |
| **CFIR domain** | **Barrier** | **Introductory question** |
| **1 Intervention charakteristics** | 1.4_Anpassbarkeit | - What changes or adjustments do you think need to be made to the intervention to make it effective to implement? - How do you think the intervention can be implemented organizationally? |
| **3 Inner Setting** | 3.3_Cultur | - How would you describe the culture in the ICU? - How do you think the culture will affect implementation? |
|  | 3.6_Compatibility | - Can you describe how the FICUS processes will be integrated into the current processes? |
|  | 3.7_Relative priority | - Are there any activities or initiatives that are top priorities for your ICU? For example, other projects? (competing) |
|  | 3.13_Available resources | - Do you have sufficient resources for implementation? |
| **4 Characteristics of individuals** | 4.2_Self-efficacy | - Do you have any idea how confident your colleagues are about implementing the intervention? |
| **5 Process** | 5.2_Opinion leaders  5.6_Stakeholders | - Who are "influential" people on the team who should be brought on board during implementation? - Are the important key players who need to be on board with the intervention? |
|  | 5.4_Champions | - Are there other people in your organization besides the official implementation support person who are committed to the intervention and who can be involved in the implementation? Champion role? |
| 1. **Implementation strategies** | | |
| **Implementation strategies** | - Leadership support - Implementation support person - Champions nursing/medical staff - Training intervention nurses - Training Team - Monthly case conferences - Individual coaching and support, feedback loops - …   *What other strategies / activities does it need? Does it cover everything?* | |
| 1. **Conclusion** | - Summary and conclusion of the interview - A written summary follows by mail | |
