# Supplementary material for: Tailored implementation of the FICUS multicomponent family support intervention in adult intensive care units: findings from a mixed methods contextual analysis
Source: BMC Health Serv Res. 2023 Dec 1;23:1339. doi: 10.1186/s12913-023-10285-1 (PMC10693161; doi:10.1186/s12913-023-10285-1)
Supplement: Supplementary file 3 — Additional file 3. Supplementary File 3. Single items of the questionnaire. [file 12913_2023_10285_MOESM3_ESM.docx]

**Supplementary File 3. Single items of the questionnaire**

| **Item** | **Median** | **IQR** |
| --- | --- | --- |
| **CFIR Inner Setting (Fernandez et al., 2018)** |  |  |
| **Culture** | 3.56 | 0.36 |
| 1. People at all levels openly talk about what is and isn’t working | 4.00 | 1.0 |
| 1. Most people in this clinic are willing to change how they do things in response to feedback from others | 4.00 | 1.00 |
| 1. It is hard to get things to change in our clinic* | 4.00 | 1.00 |
| 1. I can rely on the other people in this clinic to do their jobs well | 4.00 | 0 |
| 1. Most of the people who work in our clinic seem to enjoy their work | 4.00 | 0 |
| 1. Difficult problems are solved through face-to-face discussions | 4.00 | 1.00 |
| 1. We regularly take time to reflect on how we do things | 4.00 | 1.00 |
| 1. After trying something new, we take time to think about how it worked | 4.00 | 1.00 |
| 1. People in this clinic operate as a real team | 4.00 | 0 |
| **Culture stress** | 3.00 | 0.75 |
| 1. I am under too many pressures to do my job effectively | 2.00 | 1.00 |
| 1. Staff members often show signs of stress and strain | 4.00 | 1.00 |
| 1. The heavy workload here reduces program effectiveness | 3.00 | 2.00 |
| 1. Staff frustration is common here | 3.00 | 1.00 |
| **Culture effort** | 3.20 | 0.40 |
| 1. People in this clinic always want to preform to the best of their abilities | 4.00 | 0 |
| 1. People are enthusiastic about their work | 4.00 | 1.00 |
| 1. People in our clinic get by with doing as little as possible* | 4.00 | 1.00 |
| 1. People are prepared to make a special effort to do a good job | 4.00 | 0 |
| 1. People in this clinic do not put more effort into their work than they have to* | 4.00 | 1.00 |
| **Learning climate** | 3.80 | 0.40 |
| 1. We regularly take time to consider ways to improve how we do things | 400 | 1.00 |
| 1. People in our clinic actively seek new ways to improve how we do things | 4.00 | 0.50 |
| 1. This clinic encourages everyone to share ideas | 4.00 | 1.0 |
| 1. This clinic learns from its mistakes | 4.00 | 0 |
| 1. When we experience a problem in the clinic, we make a serious effort to figure out what’s really going on | 4.00 | 0.75 |
| **Leadership engagement** | 4.00 | 0.38 |
| 1. The clinic leadership makes sure that we have the time and space necessary to discuss changes to improve care | 4.00 | 1.00 |
| 1. Leadership in this clinic creates an environment where things can be accomplished | 4.00 | 0 |
| 1. Clinic leadership promotes an environment that is an enjoyable place to work | 4.00 | 0.50 |
| 1. Leadership strongly supports clinic change efforts | 4.00 | 0.50 |
| **Available resources** | 3.71 | 1.04 |
| 1. In general, when there is agreement that change needs to happen in the clinic, we have the necessary support in terms of: budget or financial resources | 3.00 | 1.00 |
| 1. In general, when there is agreement that change needs to happen in the clinic, we have the necessary support in terms of: training | 4.00 | 1.00 |
| 1. In general, when there is agreement that change needs to happen in the clinic, we have the necessary support in terms of: staffing | 3.00 | 2.00 |
| 1. The following are available to make the FICUS intervention work in our clinic: equipment and materials | 4.00 | 1.00 |
| 1. The following are available to make the FICUS intervention work in our clinic: patient awareness / need | 3.00 | 1.00 |
| 1. The following are available to make the FICUS intervention work in our clinic: provider buy-in | 4.00 | 1.00 |
| 1. The following are available to make the FICUS intervention work in our clinic: intervention team | 4.00 | 1.00 |
| **ORIC - Organizational Readiness for Implementing Change** | 3.58 | 0.79 |
| 1. People who work here feel confident that the organization can get people invested in implementing the FICUS intervention | 4.00 | 1.00 |
| 1. People who work here are committed to implementing the FICUS intervention | 4.00 | 1.00 |
| 1. People who work here feel confident that they can keep track of progress in implementing the FICUS intervention | 3.50 | 1.00 |
| 1. People who work here will do whatever it takes to implement the FICUS intervention delivery | 4.00 | 1.00 |
| 1. People who work here feel confident that the organization can support people as they adjust to the FICUS intervention | 4.00 | 1.00 |
| 1. People who work here want to implement the FICUS intervention | 4.00 | 1.00 |
| 1. People who work here feel confident that they can keep the momentum going in implementation of the FICUS intervention | 4.00 | 1.00 |
| 1. People who work here feel confident that they can handle the challenges that might arise in implementing the FICUS intervention | 3.50 | 1.00 |
| 1. People who work here are determined to implement the FICUS intervention | 4.00 | 1.00 |
| 1. People who work here feel confident that they can coordinate tasks so that implementation goes smoothly | 3.50 | 1.00 |
| 1. People who work here are motivated to implement the FICUS intervention | 4.00 | 0.25 |
| 1. People who work here feel confident that they can manage the politics of implementing the FICUS intervention | 3.00 | 1.00 |

Abbreviation: Standard Deviation = SD, *Indicates a reverse scored item
